# Dr. Newton M. Collins

**Published:** 1912-07

**Authors:** 


Dr. Newton M. Collins of Rochester, died June 22, aged 52. He was born at Rose, Wayne Co., N. Y., graduated at the Hahnemann Medical School of Philadelphia, 1883 and had since practiced in Rochester. He was surgeon to the Rochester Homeopathic Hospital and consulting surgeon to the Batavia Hospital. Besides filling many minor positions of honor, he had been president of the State Homeopathic Society. He had recently purchased the Sargent residence on East Avenue and was engaged in fitting it up as a surgical office, when seized with his last illness. Ten weeks ago, he developed typhoid fever and was apparently convalescent when emergent symptoms required an operation. This revealed a rupture of the right common iliac artery, at the site of an aneurysm. He died the same day. Dr. Collins was a progressive surgeon, in close personal touch with the Mayos of Rochester, Minn., and active in professional interests. His death is mourned as a personal loss by his colleagues.

